# 1-[(Pyrrolidin-1-yl)(*p*-tol­yl)meth­yl]naphthalen-2-ol

**DOI:** 10.1107/S1600536808028493

**Published:** 2008-09-13

**Authors:** Chuanwei Wan, Hong Zhao

**Affiliations:** a Ordered Matter Science Research Center, College of Chemistry and Chemical Engineering, Southeast University, Nanjing 210096, People’s Republic of China

## Abstract

In the title compound, C_22_H_23_NO, the dihedral angle between the naphthyl ring system and the benzene ring is 73.32 (6)°. An intra­molecular O—H⋯N hydrogen bond stabilizes the mol­ecular conformation. In the crystal structure, mol­ecules are linked by C—H⋯π inter­actions, resulting in zigzag chains parallel to the [10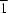] direction.

## Related literature

For general background on the chemistry of naphthalen-2-ol derivatives, see: Szatmari & Fulop (2004 ▶); Zhao & Sun (2005 ▶). For puckering and asymmetry parameters, see: Cremer & Pople (1975 ▶); Nardelli (1983 ▶).
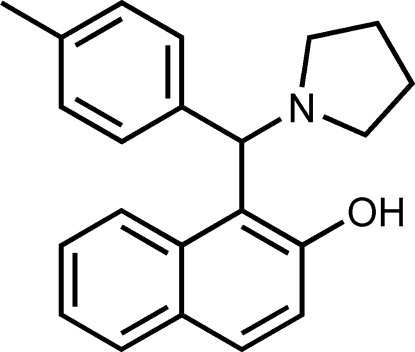


## Experimental

### 

#### Crystal data

C_22_H_23_NO
*M* *_r_* = 317.41
Monoclinic, 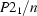

*a* = 10.3467 (18) Å
*b* = 16.055 (3) Å
*c* = 11.252 (2) Å
β = 106.810 (8)°
*V* = 1789.2 (6) Å^3^
*Z* = 4
Mo *K*α radiation
μ = 0.07 mm^−1^
*T* = 293 (2) K
0.25 × 0.22 × 0.20 mm

#### Data collection

Rigaku SCXmini diffractometer
Absorption correction: multi-scan (*CrystalClear*; Rigaku, 2005 ▶) *T*_min_ = 0.963, *T*_max_ = 0.989
18171 measured reflections
4086 independent reflections
2547 reflections with *I* > 2σ(*I*)
*R*_int_ = 0.058

#### Refinement

*R*[*F* ^2^ > 2σ(*F* ^2^)] = 0.069
*wR*(*F* ^2^) = 0.190
*S* = 1.06
4086 reflections
219 parameters
H-atom parameters constrained
Δρ_max_ = 0.25 e Å^−3^
Δρ_min_ = −0.20 e Å^−3^

### 

Data collection: *CrystalClear* (Rigaku, 2005 ▶); cell refinement: *CrystalClear*; data reduction: *CrystalClear*; program(s) used to solve structure: *SHELXS97* (Sheldrick, 2008 ▶); program(s) used to refine structure: *SHELXL97* (Sheldrick, 2008 ▶); molecular graphics: *SHELXTL/PC* (Sheldrick, 2008 ▶); software used to prepare material for publication: *SHELXTL/PC*.

## Supplementary Material

Crystal structure: contains datablocks I, global. DOI: 10.1107/S1600536808028493/rz2244sup1.cif

Structure factors: contains datablocks I. DOI: 10.1107/S1600536808028493/rz2244Isup2.hkl

Additional supplementary materials:  crystallographic information; 3D view; checkCIF report

## Figures and Tables

**Table 1:** Hydrogen-bond geometry (Å, °)

*D*—H⋯*A*	*D*—H	H⋯*A*	*D*⋯*A*	*D*—H⋯*A*
O1—H1⋯N1	0.82	1.88	2.600 (3)	145
C18—H18⋯*Cg*1^i^	0.93	2.66	3.588 (8)	173

Symmetry code: (i) 
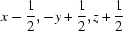
. *Cg*1 is the centroid of the C5–C10 benzene ring.

**Fig. 1.**
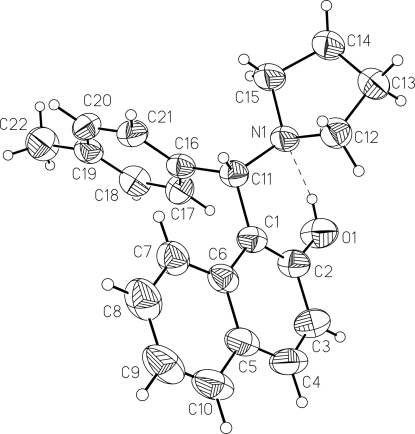
The structure of the title compound, showing the atom-numbering scheme. Displacement ellipsoids are drawn at the 50% probability level. The intramolecular O—H···N hydrogen bond is indicated by a dashed line.

## supplementary crystallographic information

### Comment

Compounds derived from naphthalen-2-ol have been of great interest in organic chemistry (Szatmari & Fulop, 2004; Zhao & Sun, 2005). We report here the crystal structure of the title compound (Fig. 1).

Bond lengths and angles in the title compound have normal values. The dihedral angle between the naphthyl and phenyl rings is 73.32 (6)°. The pyrrolidine ring adopts a twist conformation, as indicated by the puckering parameters (*q*_2_ = 0.401 (2) Å and φ = 169.2 (4)°; Cremer & Pople, 1975) and the small value of the displacement asymmetry parameter (Δ*C*_2_(C14) = 0.0301 (10)°; Nardelli, 1983). The molecular conformation is stabilized by a strong intramolecular O—H···N hydrogen bond (Table 1). In the crystal packing, molecules are linked through C—H···π interactions (Table 1) to form zig zag chains running along the [1 0 -1] direction.

### Experimental

A dry 50 ml flask was charged with benzaldehyde (10 mmol), naphthalen-2-ol (10 mmol), and pyrrolidine (10 mmol). The mixture was stirred at 100°C for 10 h then ethanol (15 ml) was added. After heating under reflux for 30 minutes, the precipitate was filtrated off and washed 3 times with ethanol to give the title compound. Single crystals suitable for X-ray analysis were obtained by slow evaporation of a dichloromethane solution.

### Refinement

All hydrogen atoms were calculated geometrically, with C—H = 0.93-0.98 Å, O—H= 0.82 Å, and refined as riding with *U*_iso_(H) = 1.2*U*_eq_(C) or 1.2*U*_eq_(C, O) for methyl and hydroxy hydrogen atoms.

### Crystal data

**Table:**

C_22_H_23_NO	*F*(000) = 680
*M**_r_* = 317.41	*D*_x_ = 1.178 Mg m^−^^3^
Monoclinic, *P*2_1_/*n*	Mo *K*α radiation, λ = 0.71073 Å
Hall symbol: -P 2yn	Cell parameters from 3280 reflections
*a* = 10.3467 (18) Å	θ = 2.3–27.4°
*b* = 16.055 (3) Å	µ = 0.07 mm^−^^1^
*c* = 11.252 (2) Å	*T* = 293 K
β = 106.810 (8)°	Prism, colourless
*V* = 1789.2 (6) Å^3^	0.25 × 0.22 × 0.20 mm
*Z* = 4	

### Data collection

**Table:**

Rigaku SCXmini diffractometer	4086 independent reflections
Radiation source: fine-focus sealed tube	2547 reflections with *I* > 2σ(*I*)
graphite	*R*_int_ = 0.058
Detector resolution: 13.6612 pixels mm^-1^	θ_max_ = 27.4°, θ_min_ = 2.3°
ω scans	*h* = −13→13
Absorption correction: multi-scan (CrystalClear; Rigaku, 2005)	*k* = −20→20
*T*_min_ = 0.963, *T*_max_ = 0.989	*l* = −14→14
18171 measured reflections	

### Refinement

**Table:**

Refinement on *F*^2^	Primary atom site location: structure-invariant direct methods
Least-squares matrix: full	Secondary atom site location: difference Fourier map
*R*[*F*^2^ > 2σ(*F*^2^)] = 0.069	Hydrogen site location: inferred from neighbouring sites
*wR*(*F*^2^) = 0.190	H-atom parameters constrained
*S* = 1.06	*w* = 1/[σ^2^(*F*_o_^2^) + (0.0911*P*)^2^ + 0.1709*P*] where *P* = (*F*_o_^2^ + 2*F*_c_^2^)/3
4086 reflections	(Δ/σ)_max_ = 0.005
219 parameters	Δρ_max_ = 0.25 e Å^−^^3^
0 restraints	Δρ_min_ = −0.20 e Å^−^^3^

### Special details

**Table:**

Geometry. All e.s.d.'s (except the e.s.d. in the dihedral angle between two l.s. planes) are estimated using the full covariance matrix. The cell e.s.d.'s are taken into account individually in the estimation of e.s.d.'s in distances, angles and torsion angles; correlations between e.s.d.'s in cell parameters are only used when they are defined by crystal symmetry. An approximate (isotropic) treatment of cell e.s.d.'s is used for estimating e.s.d.'s involving l.s. planes.
Refinement. Refinement of *F*^2^ against ALL reflections. The weighted *R*-factor *wR* and goodness of fit *S* are based on *F*^2^, conventional *R*-factors *R* are based on *F*, with *F* set to zero for negative *F*^2^. The threshold expression of *F*^2^ > σ(*F*^2^) is used only for calculating *R*-factors(gt) *etc*. and is not relevant to the choice of reflections for refinement. *R*-factors based on *F*^2^ are statistically about twice as large as those based on *F*, and *R*- factors based on ALL data will be even larger.

### Fractional atomic coordinates and isotropic or equivalent isotropic displacement parameters (Å^2^)

**Table:**

	*x*	*y*	*z*	*U*_iso_*/*U*_eq_	
C1	0.2106 (2)	0.17738 (14)	0.94453 (19)	0.0419 (5)	
C2	0.2318 (2)	0.10314 (14)	1.0087 (2)	0.0484 (6)	
C3	0.3641 (2)	0.07349 (17)	1.0645 (2)	0.0593 (7)	
H3	0.3762	0.0225	1.1056	0.071*	
C4	0.4730 (2)	0.11799 (18)	1.0592 (2)	0.0603 (7)	
H4	0.5591	0.0970	1.0959	0.072*	
C5	0.4578 (2)	0.19606 (16)	0.9986 (2)	0.0511 (6)	
C6	0.3253 (2)	0.22609 (14)	0.9413 (2)	0.0437 (5)	
C7	0.3143 (2)	0.30541 (15)	0.8828 (2)	0.0526 (6)	
H7	0.2292	0.3276	0.8455	0.063*	
C8	0.4263 (3)	0.34974 (18)	0.8801 (3)	0.0680 (8)	
H8	0.4161	0.4014	0.8410	0.082*	
C9	0.5550 (3)	0.3189 (2)	0.9349 (3)	0.0713 (8)	
H9	0.6302	0.3495	0.9317	0.086*	
C10	0.5714 (2)	0.2439 (2)	0.9932 (3)	0.0654 (8)	
H10	0.6579	0.2236	1.0299	0.078*	
C11	0.0689 (2)	0.20844 (12)	0.87783 (19)	0.0396 (5)	
H11	0.0738	0.2388	0.8037	0.048*	
C12	0.0091 (2)	0.08850 (15)	0.7372 (2)	0.0512 (6)	
H12A	0.0886	0.0544	0.7710	0.061*	
H12B	0.0248	0.1249	0.6740	0.061*	
C13	−0.1145 (2)	0.03501 (16)	0.6848 (2)	0.0603 (7)	
H13A	−0.1071	−0.0171	0.7298	0.072*	
H13B	−0.1269	0.0231	0.5977	0.072*	
C14	−0.2303 (2)	0.08716 (16)	0.7016 (3)	0.0617 (7)	
H14A	−0.2831	0.0555	0.7445	0.074*	
H14B	−0.2890	0.1048	0.6218	0.074*	
C15	−0.1655 (2)	0.16206 (16)	0.7785 (2)	0.0563 (7)	
H15A	−0.1704	0.2107	0.7263	0.068*	
H15B	−0.2098	0.1745	0.8415	0.068*	
C16	0.01458 (19)	0.26802 (13)	0.95666 (19)	0.0395 (5)	
C17	0.0117 (2)	0.24824 (14)	1.0757 (2)	0.0477 (5)	
H17	0.0454	0.1972	1.1100	0.057*	
C18	−0.0407 (2)	0.30347 (15)	1.1441 (2)	0.0528 (6)	
H18	−0.0428	0.2884	1.2233	0.063*	
C19	−0.0899 (2)	0.38058 (15)	1.0975 (2)	0.0479 (6)	
C20	−0.0865 (2)	0.39997 (14)	0.9788 (2)	0.0493 (6)	
H20	−0.1189	0.4514	0.9450	0.059*	
C21	−0.0361 (2)	0.34482 (14)	0.9096 (2)	0.0458 (5)	
H21	−0.0362	0.3595	0.8296	0.055*	
C22	−0.1439 (3)	0.44099 (18)	1.1736 (3)	0.0712 (8)	
H22A	−0.1290	0.4193	1.2560	0.107*	
H22B	−0.2389	0.4488	1.1358	0.107*	
H22C	−0.0982	0.4934	1.1776	0.107*	
N1	−0.02391 (16)	0.13698 (11)	0.83579 (16)	0.0414 (4)	
O1	0.13049 (17)	0.05359 (11)	1.02090 (17)	0.0641 (5)	
H1	0.0585	0.0700	0.9744	0.096*	

### Atomic displacement parameters (Å^2^)

**Table:**

	*U*^11^	*U*^22^	*U*^33^	*U*^12^	*U*^13^	*U*^23^
C1	0.0345 (11)	0.0496 (13)	0.0407 (12)	−0.0007 (9)	0.0094 (9)	−0.0015 (9)
C2	0.0431 (12)	0.0535 (14)	0.0463 (13)	−0.0024 (10)	0.0091 (10)	0.0064 (10)
C3	0.0518 (14)	0.0671 (17)	0.0532 (15)	0.0093 (13)	0.0057 (12)	0.0106 (12)
C4	0.0392 (12)	0.0837 (19)	0.0513 (15)	0.0098 (12)	0.0024 (11)	−0.0049 (13)
C5	0.0368 (11)	0.0684 (16)	0.0465 (13)	−0.0041 (11)	0.0097 (10)	−0.0150 (11)
C6	0.0373 (11)	0.0525 (13)	0.0425 (12)	−0.0048 (9)	0.0135 (9)	−0.0096 (10)
C7	0.0464 (13)	0.0510 (14)	0.0629 (16)	−0.0094 (11)	0.0197 (11)	−0.0057 (11)
C8	0.0629 (17)	0.0617 (17)	0.086 (2)	−0.0203 (13)	0.0317 (15)	−0.0078 (14)
C9	0.0527 (16)	0.085 (2)	0.081 (2)	−0.0297 (15)	0.0271 (15)	−0.0208 (16)
C10	0.0368 (12)	0.094 (2)	0.0635 (17)	−0.0096 (13)	0.0123 (12)	−0.0234 (15)
C11	0.0361 (10)	0.0415 (12)	0.0407 (12)	−0.0038 (9)	0.0103 (9)	0.0045 (9)
C12	0.0431 (12)	0.0561 (15)	0.0543 (14)	−0.0004 (10)	0.0143 (11)	−0.0072 (11)
C13	0.0517 (14)	0.0602 (16)	0.0629 (16)	−0.0037 (12)	0.0070 (12)	−0.0137 (12)
C14	0.0407 (12)	0.0626 (16)	0.0748 (18)	−0.0043 (11)	0.0055 (12)	−0.0118 (13)
C15	0.0343 (12)	0.0621 (16)	0.0671 (16)	0.0008 (10)	0.0062 (11)	−0.0113 (12)
C16	0.0313 (10)	0.0451 (12)	0.0409 (12)	−0.0050 (9)	0.0086 (9)	0.0021 (9)
C17	0.0527 (13)	0.0446 (13)	0.0473 (13)	0.0006 (10)	0.0168 (11)	0.0070 (10)
C18	0.0560 (14)	0.0626 (16)	0.0433 (13)	−0.0062 (12)	0.0198 (11)	0.0010 (11)
C19	0.0335 (11)	0.0544 (14)	0.0543 (15)	−0.0033 (10)	0.0105 (10)	−0.0063 (11)
C20	0.0415 (12)	0.0446 (13)	0.0584 (15)	0.0059 (10)	0.0088 (11)	0.0044 (10)
C21	0.0411 (11)	0.0499 (14)	0.0440 (13)	0.0009 (10)	0.0085 (10)	0.0067 (10)
C22	0.0627 (17)	0.077 (2)	0.0782 (19)	0.0021 (14)	0.0275 (15)	−0.0171 (15)
N1	0.0309 (9)	0.0458 (10)	0.0463 (11)	−0.0024 (7)	0.0093 (8)	−0.0028 (8)
O1	0.0508 (10)	0.0634 (12)	0.0733 (13)	−0.0053 (8)	0.0102 (9)	0.0243 (9)

### Geometric parameters (Å, °)

**Table:**

C1—C2	1.378 (3)	C12—H12B	0.9700
C1—C6	1.430 (3)	C13—C14	1.518 (3)
C1—C11	1.525 (3)	C13—H13A	0.9700
C2—O1	1.354 (3)	C13—H13B	0.9700
C2—C3	1.413 (3)	C14—C15	1.519 (3)
C3—C4	1.351 (4)	C14—H14A	0.9700
C3—H3	0.9300	C14—H14B	0.9700
C4—C5	1.414 (4)	C15—N1	1.476 (3)
C4—H4	0.9300	C15—H15A	0.9700
C5—C10	1.420 (3)	C15—H15B	0.9700
C5—C6	1.420 (3)	C16—C21	1.384 (3)
C6—C7	1.423 (3)	C16—C17	1.385 (3)
C7—C8	1.367 (3)	C17—C18	1.383 (3)
C7—H7	0.9300	C17—H17	0.9300
C8—C9	1.387 (4)	C18—C19	1.383 (3)
C8—H8	0.9300	C18—H18	0.9300
C9—C10	1.357 (4)	C19—C20	1.381 (3)
C9—H9	0.9300	C19—C22	1.504 (3)
C10—H10	0.9300	C20—C21	1.378 (3)
C11—N1	1.483 (2)	C20—H20	0.9300
C11—C16	1.518 (3)	C21—H21	0.9300
C11—H11	0.9800	C22—H22A	0.9600
C12—N1	1.474 (3)	C22—H22B	0.9600
C12—C13	1.511 (3)	C22—H22C	0.9600
C12—H12A	0.9700	O1—H1	0.8200
			
C2—C1—C6	118.6 (2)	C14—C13—H13A	110.9
C2—C1—C11	121.71 (19)	C12—C13—H13B	110.9
C6—C1—C11	119.71 (19)	C14—C13—H13B	110.9
O1—C2—C1	123.4 (2)	H13A—C13—H13B	108.9
O1—C2—C3	115.8 (2)	C13—C14—C15	105.88 (19)
C1—C2—C3	120.8 (2)	C13—C14—H14A	110.6
C4—C3—C2	121.0 (2)	C15—C14—H14A	110.6
C4—C3—H3	119.5	C13—C14—H14B	110.6
C2—C3—H3	119.5	C15—C14—H14B	110.6
C3—C4—C5	120.8 (2)	H14A—C14—H14B	108.7
C3—C4—H4	119.6	N1—C15—C14	104.57 (18)
C5—C4—H4	119.6	N1—C15—H15A	110.8
C4—C5—C10	121.5 (2)	C14—C15—H15A	110.8
C4—C5—C6	118.6 (2)	N1—C15—H15B	110.8
C10—C5—C6	120.0 (3)	C14—C15—H15B	110.8
C5—C6—C7	116.8 (2)	H15A—C15—H15B	108.9
C5—C6—C1	120.2 (2)	C21—C16—C17	117.6 (2)
C7—C6—C1	123.0 (2)	C21—C16—C11	120.06 (19)
C8—C7—C6	121.4 (2)	C17—C16—C11	122.37 (19)
C8—C7—H7	119.3	C18—C17—C16	120.8 (2)
C6—C7—H7	119.3	C18—C17—H17	119.6
C7—C8—C9	121.0 (3)	C16—C17—H17	119.6
C7—C8—H8	119.5	C19—C18—C17	121.6 (2)
C9—C8—H8	119.5	C19—C18—H18	119.2
C10—C9—C8	120.1 (2)	C17—C18—H18	119.2
C10—C9—H9	120.0	C20—C19—C18	117.3 (2)
C8—C9—H9	120.0	C20—C19—C22	121.5 (2)
C9—C10—C5	120.7 (3)	C18—C19—C22	121.2 (2)
C9—C10—H10	119.6	C21—C20—C19	121.4 (2)
C5—C10—H10	119.6	C21—C20—H20	119.3
N1—C11—C16	111.04 (16)	C19—C20—H20	119.3
N1—C11—C1	110.22 (16)	C20—C21—C16	121.3 (2)
C16—C11—C1	112.58 (17)	C20—C21—H21	119.4
N1—C11—H11	107.6	C16—C21—H21	119.4
C16—C11—H11	107.6	C19—C22—H22A	109.5
C1—C11—H11	107.6	C19—C22—H22B	109.5
N1—C12—C13	103.87 (18)	H22A—C22—H22B	109.5
N1—C12—H12A	111.0	C19—C22—H22C	109.5
C13—C12—H12A	111.0	H22A—C22—H22C	109.5
N1—C12—H12B	111.0	H22B—C22—H22C	109.5
C13—C12—H12B	111.0	C12—N1—C15	103.41 (17)
H12A—C12—H12B	109.0	C12—N1—C11	112.27 (16)
C12—C13—C14	104.3 (2)	C15—N1—C11	113.43 (17)
C12—C13—H13A	110.9	C2—O1—H1	109.5

### Hydrogen-bond geometry (Å, °)

**Table:**

*D*—H···*A*	*D*—H	H···*A*	*D*···*A*	*D*—H···*A*
O1—H1···N1	0.82	1.88	2.600 (3)	145
C18—H18···Cg1^i^	0.93	2.66	3.588 (8)	173

Symmetry codes: (i) *x*−1/2, −*y*+1/2, *z*+1/2.
